# Alpha oscillations reflect similar mapping mechanisms for localizing touch on hands and tools

**DOI:** 10.1016/j.isci.2024.109092

**Published:** 2024-02-02

**Authors:** Cécile Fabio, Romeo Salemme, Alessandro Farnè, Luke E. Miller

**Affiliations:** 1 Integrative Multisensory Perception Action & Cognition Team of the Lyon Neuroscience Research, Center INSERM U1028 CNRS U5292 University of Lyon 1, Lyon, France; 2 Hospices Civils de Lyon, Neuro-immersion, Lyon, France; 3 Donders Institute for Brain, Cognition and Behaviour, Nijmegen, the Netherlands; 4 Department for Cognitive Neuroscience, Faculty of Biology, Bielefeld University, Bielefeld, Germany

**Keywords:** Physics, Neuroscience, Engineering

## Abstract

It has been suggested that our brain re-uses body-based computations to localize touch on tools, but the neural implementation of this process remains unclear. Neural oscillations in the alpha and beta frequency bands are known to map touch on the body in external and skin-centered coordinates, respectively. Here, we pinpointed the role of these oscillations during tool-extended sensing by delivering tactile stimuli to either participants’ hands or the tips of hand-held rods. To disentangle brain responses related to each coordinate system, we had participants’ hands/tool tips crossed or uncrossed at their body midline. We found that midline crossing modulated alpha (but not beta) band activity similarly for hands and tools, also involving a similar network of cortical regions. Our findings strongly suggest that the brain uses similar oscillatory mechanisms for mapping touch on the body and tools, supporting the idea that body-based neural processes are repurposed for tool use.

## Highlights

• Touch on hands and tools relies on shared oscillatory mapping mechanisms
• Alpha rhythm reflects external spatial coding for touch on hands and tools

## Graphical abstract

**Figure:**
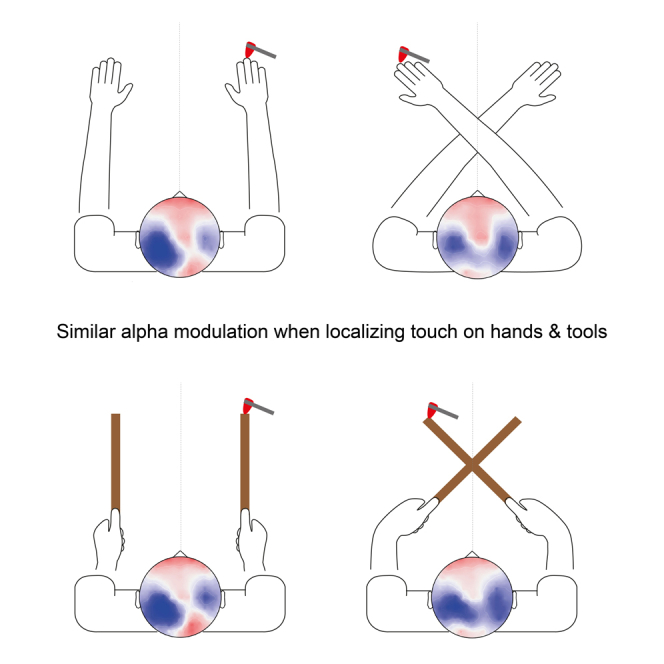

## Introduction

Tools allow us to extend our physical body, therefore amplifying our sensorimotor abilities. It is theorized that tools become incorporated into a neural representation of our body^1^^,^^2^^,^^3^ as tool use notably alters motor kinematics, representation of body metrics,^4^^,^^5^^,^^6^^,^^7^^,^^8^ and representation of space around the upper limb.^9^^,^^10^ Alongside these studies, Miller et al.^11^ recently found that participants can accurately localize where an object touches the surface of a hand-held tool, thus using the tool as a sensory extension of their body. These behavioral effects prompted the hypothesis that the brain repurposes body-based neural processing to control and sense with a tool. However, evidence for this hypothesis is currently limited.^12^^,^^13^^,^^14^ Here we used tool-extended tactile localization as a case study to investigate the brain repurposing of body-based neural computations during tool use.

Comparing touch localization on hands and tools requires a thorough understanding of the underlying neural computations that map touch on the body.^15^ Acting toward a touch requires transforming a representation of the location of touch on the skin (anatomical coordinates) into a represented location of touch in external space (external coordinates). This process, called tactile remapping, involves the integration of proprioceptive and cutaneous signals. Tactile remapping highlights the use of multiple spatial codes that underlie tactile localization.

At the level of neural oscillations, touch on the skin leads to a desynchronization of power in two main low-frequency bands: alpha (8–13 Hz)^16^^,^^17^^,^^18^^,^^19^^,^^20^ and beta (15–25 Hz).^18^^,^^20^^,^^21^ These frequency bands have been implicated in tactile localization within two types of computational spatial codes^22^: beta activity reflects encoding in anatomical coordinates,^23^^,^^24^^,^^25^^,^^26^ which correspond to the position of touch on the skin; alpha activity reflects encoding in external coordinates,^23^^,^^24^^,^^25^^,^^26^^,^^27^ which correspond to the position of touch in the space around the body. It is important to note that these two reference frames—anatomical and external—are egocentric, as in both cases the location of objects is coded in reference to the same observer.

Typically, the processes behind these spatial codes have been disambiguated by crossing the hands over the body midline: when crossed, the right hand (anatomical coordinates) is located in the left hemispace (external coordinates), thus creating left-right conflict that affects behavioral performance across several localization tasks.^28^^,^^29^^,^^30^ This conflict is often emphasized by attention cueing paradigms, as mapping touch using external coordinates may require orienting spatial attention.^25^^,^^31^^,^^32^ Alpha oscillations have indeed been implicated in tactile spatial attention^33^^,^^34^^,^^35^^,^^36^ and have also been shown to be affected when changing posture in a similar experimental paradigm.^25^

There is reason to believe that localizing touch on a tool may involve similar neurocomputational mechanisms. At a behavioral level, touch on hand-held tools can be localized with a similar level of accuracy as touch on the body^11^ and is similarly affected when the tips of the tools are crossed over the midline while the hands remain uncrossed.^30^^,^^37^ Our previous electroencephalogram (EEG) study revealed the involvement of alpha (but not beta) activity in tool-extended tactile localization, with sources in a network of parieto-frontal areas involved in tactile and spatial processing.^14^ However, the nature of the reference frame(s) underlying the observed alpha activity remains unclear.

To fill this gap, here we investigated whether the oscillatory mechanisms involved in localizing touch on the body are repurposed when localizing touch on a tool. To determine this, we characterized and compared the reference frames reflected in alpha- and beta-band oscillatory activity, during body-based and tool-extended tactile localization. We used EEG to record oscillatory activity of participants performing a cued tactile localization task on their hands and on hand-held tools while manipulating their posture (crossed vs. uncrossed). When tool tips were crossed over the midline, the hands holding the tools were always uncrossed, allowing us to directly compare tool-based and body-based reference frames.

## Results

The aim of the present study was to compare the oscillatory correlates of tactile spatial coding for touch on hands and hand-held tools. To do so, we administered a tactile discrimination task—which has previously been used to disentangle anatomical and external reference frames^25^^,^^26^—for stimuli applied via solenoids to either the hands or hand-held tools (50 cm wooden rods). We recorded each participant’s neural dynamics during this task with 64-channel EEG (see STAR methods).

### Similar tactile localization behavior for touch on hands and tools

**Figure 1:**
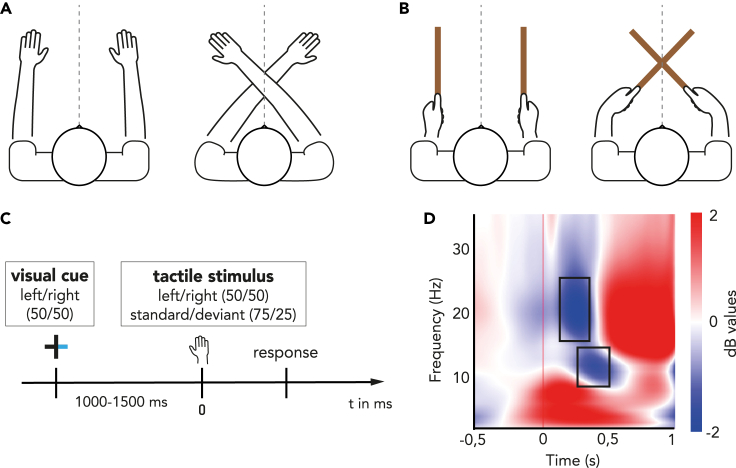
Experimental setup and paradigm Participants (n = 20) performed a tactile discrimination task for touches applied on two surfaces: (A) when applied on hands, participants hold their hands either in an uncrossed posture (left) or a crossed posture (right). (B) When applied on tools, participants hold tools either in an uncrossed posture (left) or a crossed posture (right) where only the tool tips crossed over the body midline (gray dotted line). (C) Trial structure of the tactile discrimination task: each trial started with the central cross blinking, followed by a spatial cue (half of the cross briefly turning blue) to indicate which side of space (left or right, equal probability) participants had to attend to. After a variable delay, tactile stimulation (here corresponding to time zero) was applied on participants’ right or left finger, or on the tip of the right or left rod, independently of the cued side. Tactile stimuli were either frequent standard stimuli (“single touch”, probability of 0.75), or rare deviant stimuli (“double touch”, probability of 0.25). Participants had to respond as fast and accurately as possible to rare tactile deviants presented to the cued side, and to ignore standard stimuli at the attended side, as well as all stimuli presented to the other side. (D) Total oscillatory activity of the post-stimulation period over contralateral somatosensory cortex (electrode C3) obtained from time-frequency decomposition using complex Morlet wavelets. Modulations are displayed as compared relative to baseline (−500 to −100 ms). Selected time windows for analysis are represented by gray rectangle for each frequency band: 250–500 ms for alpha and 150–300 ms for beta.

We administered a tactile discrimination task (Figures 1A–1C) that implicitly required participants (n = 20) to process the location of touch. In the task (Figure 1C), participants discriminated between standard and deviant tactile stimuli, which was either a single tap or a double tap, respectively. At the start of each trial, they were cued to pay attention to one side of external space (left or right of the midline) before receiving tactile stimulation on either hand/tool. They were then required to indicate (via a foot pedal) the presence of a deviant stimulus within the cued location of external space, ignoring the standard stimulus and all stimuli within the uncued side of space. Importantly, the posture of the hands or tools (Figures 1A and 1B) during the task was either uncrossed or crossed across the midline. This postural manipulation allowed us to disentangle the involvement of brain oscillations in different reference frames transformations. It is crucial to note that, when touch was applied to the tools, only the tool posture changed; the hand posture remained uncrossed (Figure 1B).

Participants performance on this task was near ceiling and within range of what has been found previously^11^^,^^14^ (Table 1). The hit rate for stimuli was high (>95%) for all conditions and was independent of the touched surface (hand, tool) or the posture (crossed, uncrossed). The reaction times were also unaffected by the touched surface or the posture. Participants’ performance was thus comparable, whether touch was delivered on the hands or tools, consistent with prior findings that tactile localization is similar for touch on a body part and a hand-held tool.^11^

**Table 1:** Behavioral performance on the tactile discrimination task

Condition	Hit rate (%)	Median RT (ms)
Hand uncrossed	96.9 (±1.67)	438
Hand crossed	96.6 (±2.24)	443
Tool uncrossed	96.3 (±2.25)	476
Tool crossed	95.8 (±2.17)	458

The hit rate corresponds to the percentage of correctly identified deviant stimuli. RT: reaction time in milliseconds. There was no significant difference between the hand and the tool condition, as well as between uncrossed and crossed conditions.

### Similar oscillatory correlates for tactile localization on hands and tools

The main aim of our study was to compare the oscillatory mechanisms underlying the mapping of touch on hands and hand-held tools. We placed a particular focus on activity within the alpha (8–13 Hz) and beta (15–25 Hz) bands post-stimulus (Figure 1D), as these have been implicated in the mapping of touch in external and anatomical reference frames,^23^^,^^24^^,^^26^^,^^26^^,^^27^ respectively. To extract the oscillatory power within each band, time-frequency decomposition was applied to the EEG recording of the post-touch period of the standard stimuli (see STAR methods). To isolate processing of touch occurring mainly within an external reference frame, we reorganized the electrodes channels to ipsi- and contralateral recording sites relative to the touched hand or tool.^23^^,^^25^

Our analyses focused on the effects of three factors on the oscillatory power of each frequency band: the surface touched (Surface: hand, tool), the posture of the surface (Posture: crossed, uncrossed), and whether the tactile stimulus was within the external locus of attention (Attention: attended, unattended). Given our aim to compare the oscillatory correlates for localizing touch on hands and on tools—with a specific focus on the external spatial coding of touch—the statistical contrasts containing the factor Surface are of particular interest. This includes one three-way interaction (Attention x Posture x Surface), two two-way interactions (Attention x Surface, Posture x Surface), and a main effect of Surface.

We first determined whether there were statistically significant differences in the oscillatory power of alpha and beta for the aforementioned contrasts. Significant differences in these contrasts would indicate that tactile localization modulated oscillatory power differently between the hand and the tool. Using a cluster-based permutation test (CBPT, α-range = 0.05), we found no significant interactions or main effects with the factor Surface in either the alpha or beta band (Table 2, CBPT: p > 0.05) suggesting similar modulations of oscillatory power when localizing touch on the hand and on the tool.

**Table 2:** Contrasts performed on scalp topographies of mean oscillatory power

Contrasts	Frequency band	p-value	
Posture	Alpha	0.316	*/*
	Beta	*/*	*/*
Attention	Alpha	0.012	*/*
	Beta	*/*	0.046
Surface	Alpha	0.276	0.346
	Beta	0.164	0.359
Posture × Surface	Alpha	*/*	*/*
	Beta	*/*	*/*
Attention × Surface	Alpha	*/*	*/*
	Beta	0.342	*/*
Attention × Posture	Alpha	0.009	0.044
	Beta	0.194	*/*
Attention × Posture × Surface	Alpha	0.242	*/*
	Beta	*/*	*/*

CBPT, p-value of all clusters found. The p-value column is divided into two columns (left and right) for clusters either found in the left or right hemisphere. ‘/’ indicates no cluster significant found.

### Spatial attention modulates tactile processing according to posture

In light of the non-significant differences between oscillations while localizing touch on hands and tools, we proceeded to investigate the surface-independent effects of posture on oscillations. A significant effect containing the factor Posture (uncrossed vs. crossed) would suggest processing related to an external reference frame. We did not observe a general main effect of Posture for alpha or beta power (Table 2, CBPT: p > 0.05).

**Figure 2:**
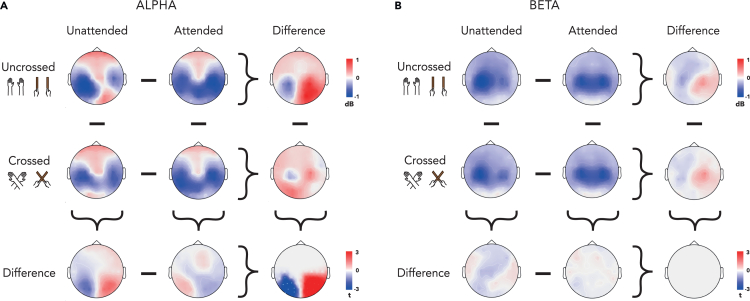
Alpha and beta activity after tactile stimulation (A) Topographies of alpha-band activity (8–13 Hz, 250 to 500 ms) in uncrossed (1st row) and crossed (2nd row) posture following unattended (1st column) and attended stimuli (2nd column). Difference topographies for attention effects in uncrossed and crossed posture (3rd column), and for posture effects following attended and unattended stimuli (3rd row). Bottom-right corner: topography of the interaction between attention and posture. (B) Topographies of beta-band activity (15-25Hz, 150 to 300ms) in uncrossed (1st row) and crossed (2nd row) posture following unattended (1st column) and attended stimuli (2nd column). Difference topographies for attention effects in uncrossed and crossed posture (3rd column), and for posture effects following attended and unattended stimuli (3rd row). Bottom-right corner: topography of the interaction between attention and posture. Data are displayed as if stimuli always occurred on the anatomically right hand or the tool held in the right hand, so that the left hemisphere is contralateral to tactile stimulation in a skin-based reference frame, independent of posture.

We did, however, observe a significant interaction effect between Attention and Posture for power in the alpha band in both hemispheres (left: p value = 0.049; right: p value = 0.006). The two significant clusters were localized above parieto-occipital channels in their respective hemisphere (Figure 2A). As can be seen in Figure 2A, touch led to widespread alpha desynchronization across centro-posterior channels, which was increased when attention was directed to the touched side (Main effect of Attention: p < 0.05, see Table 2). Crucially, we observed that crossing the surface (hand or tool) shifted the topographic distribution of alpha desynchronization: in the unattended condition, alpha desynchronization was greater over left centro-posterior electrodes when the surface was uncrossed but more uniformly spread over both hemispheres when the surface was crossed. In contrast, there was little difference in the scalp distribution between crossed and uncrossed surfaces in the attended conditions (lower panels of Figure 2A).

When looking at the interaction effect between Attention and Posture for the beta band, we observed a bilateral decrease of power over central channels that was independent of Posture (see Figure 2B). We found that the desynchronization of beta was more bilateral in the attended condition (Main effect of Attention: p < 0.05, see Table 2). However, we did not find a significant interaction between Attention and Posture in the beta band (Figure 2B).

### Similar topography of alpha desynchronization is observed for hands and tools

**Figure 3:**
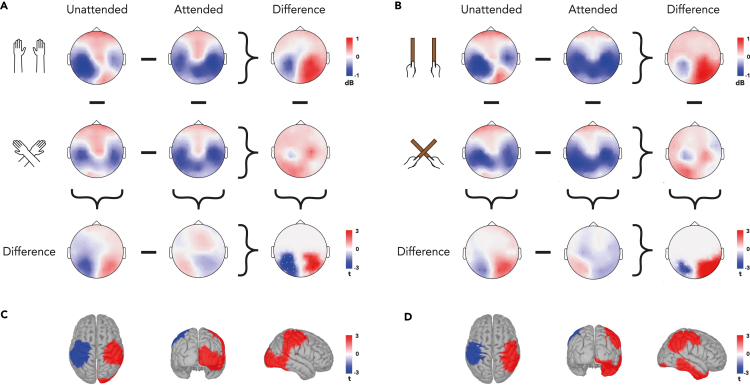
Alpha activity following tactile stimulation on the hand and on the tool (A) Topographies of alpha-band activity (8–13 Hz, 250 to 500 ms) when tactile stimuli happened on the hand, with uncrossed (1st row) and crossed (2nd row) hands following attended (1st column) and unattended (2nd column) stimuli. Different topographies for attention effects with uncrossed and crossed hands (3rd column), and for posture effects following attended and unattended stimuli (3rd row). Bottom-right corner: topography of the interaction between attention and posture. (B) Topographies of alpha-band activity (8–13 Hz, 250 to 500 ms) when tactile stimuli happened on the tool, with uncrossed (1st row) and crossed hands (2nd row) following attended (1st column) and unattended (2nd column) stimuli. Different topographies for attention effects with uncrossed and crossed tools (3rd column), and for posture effects following attended and unattended stimuli (3rd row). Bottom-right corner: topography of the interaction between attention and posture. (C) Source reconstruction of the interaction effect between attention and posture for tactile stimulation on the hand. (D) Source reconstruction of the interaction effect between attention and posture for tactile stimulation on the tool. Data are displayed as if stimuli always occurred on the anatomically right hand or tool held in the right hand, so that the left hemisphere is contralateral to tactile stimulation in a skin-based reference frame, independent of posture.

In order to further explore the localization processes for touch on hands and on tools, we then analyzed each surface separately. This was done with the purpose of confirming the presence of a similar pattern of attentional and postural modulation for each surface. Visual inspection of the scalp distribution of alpha activity for each surface reveals a striking resemblance between hand and tool (Figures 3A and 3B). For each condition, alpha power modulation is almost identical between the two surfaces, with patterns reflecting what we observed when surfaces were collapsed (Figure 2A). This underscores the inference that the neural processes underlying localizing touch on each surface are similar.

Subsequently, we calculated the interaction between Attention and Posture for each surface separately. We found a similar pattern of interaction between hand and tool: for stimulation on the hand, we obtained two clusters, one in each hemisphere (CBPT, left: p = 0.036 & right: p = 0.074). We also obtained two clusters with similar distribution when stimulation happened on the tool (CBPT, left: p = 0.195 & right: p = 0.022). While not all clusters reached statistical significance for each surface, their overall distribution corresponded well to the interaction between Attention and Posture observed when surfaces were collapsed (Figure 2A) and therefore displaying a comparable pattern of reference-frame-based oscillatory processing.

To further bolster the evidence for a similarity between the effects of external processing for touch on hands and tools, we used a Bayesian approach to compare the alpha desynchronizations in each significant cluster. For both clusters, we observed a Bayes factor greater than 3 in favor of a similarity between the alpha-based mapping mechanisms. This goes beyond the results of the aforementioned cluster-based analysis, providing positive evidence in favor of our claim that touch on hands and tools is mapped similarly in external space.

We next identified the cortical sources underlying this interaction effect for each surface. Again, we observed similar sources for localization on hands and tools: The interaction effect was significant throughout sensorimotor regions, including the primary somatosensory, primary motor cortices, and posterior parietal regions contralateral to the stimulated hand (Figure 3C, p = 0.046) and tool (Figure 3D, p = 0.024). In the hemisphere ipsilateral to the stimulated hand, cortical sources included the same sensorimotor fronto-parietal regions as well as the occipito-temporal cortex (Figure 3C, p = 0.006). The interaction effect in the hemisphere ipsilateral to the stimulated tool also spread over the same fronto-parietal regions (Figure 3D, p = 0.006) but, in contrast with the hand, also included a larger portion of the temporal cortices (Figure 3D, p = 0.019). In general, nearly identical sources were found for mapping touch on either hands or tools.

### Beta desynchronization reflects attention-driven coding of touch in anatomical coordinates

The previous analysis was aimed at isolating spatial coding in external coordinates. We thus next aimed to identify oscillations related to spatial coding in anatomical coordinates, for hands and tools. To this aim, we first reorganized the electrodes channels to ipsi- and contralateral recording sites relative to the hemispace where the touch happened. As such, all trials were set in reference to the right side of space being touched. This allowed to focus on the anatomical coding of touch by mapping the contrast of “Posture” onto different sides of anatomical space. We can thus conceptualize touch in the uncrossed condition as corresponding to right anatomical space and the crossed condition as left anatomical space.

**Figure 4:**
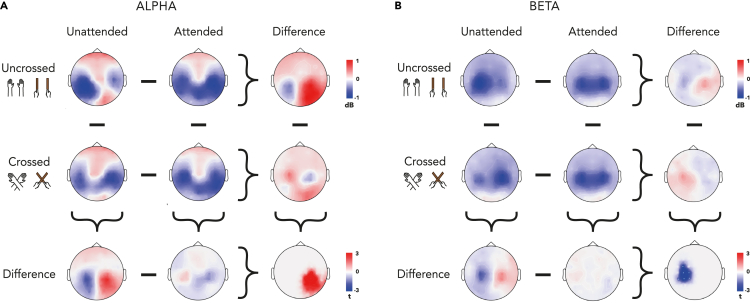
Alpha and beta activity after tactile stimulation in the right hemispace (A) Topographies of alpha-band activity (8–13 Hz, 250 to 500 ms) in uncrossed (1st row) and crossed (2nd row) posture following unattended (1st column) and attended stimuli (2nd column). Different topographies for attention effects in uncrossed and crossed posture (3rd column), and for posture effects following attended and unattended stimuli (3rd row). Bottom-right corner: topography of the interaction between attention and posture. (B) Topographies of beta-band activity (15–25 Hz, 150 to 300 ms) in uncrossed (1st row) and crossed (2nd row) posture following unattended (1st column) and attended stimuli (2nd column). Different topographies for attention effects in uncrossed and crossed posture (3rd column), and for posture effects following attended and unattended stimuli (3rd row). Bottom-right corner: topography of the interaction between attention and posture. Data are displayed as if stimuli always occurred on the hand or tool localized in the right hemispace, independent of posture.

As in our previous analyses, a CBPT revealed no significant interactions with the factor Surface in either the beta or the alpha-band in this new configuration. We observed again a significant interaction effect between Attention and Posture for power in the alpha band (Figure 4A, p = 0.012). However, this was specific to the right hemisphere and shifted slightly more centrally than the previous analyses (see Figure 2).

Unlike the previous analysis, we now found significant results for the distribution of beta desynchronization (see Figure 4B). We again observed a bilateral decrease of power over central channels in the attended condition that was independent of Posture. Beta desynchronization was more bilateral in the attended condition, being stronger in the left hemisphere for the uncrossed condition but stronger for the right hemisphere in the crossed condition. We did find a significant interaction between Attention and Posture in the beta band above central electrodes in the right hemisphere (Figure 4B, p = 0.042). This result highlights the role of beta in the attention-driven coding of touch in anatomical coordinates.

We then analyzed each Surface separately for the beta activity. These analyses, which can be found in the supplementary materials, demonstrated again a similarity between oscillatory activities involved in processing touch on hands and tools. In addition, they further bolstered the suggestions that brain activity in the beta band plays a relatively minor role, if anything, in the coding of touch in external coordinates, being more important for the anatomical reference frame coding of touch.

## Discussion

The present study was designed to identify the neural mechanisms used for localizing tactile stimuli delivered on hand-held tools and to compare them with those typically used when localizing tactile stimuli applied on hands. To this end, we used EEG in a cued tactile discrimination task whereby we manipulated hand and tool posture (crossed vs. uncrossed). Our first analysis approach emphasized differences within the external reference frame whereas the second approach highlighted differences within the anatomical reference frame. For both approaches, we found a remarkable similarity of alpha and beta power modulation following touch between the two surfaces. Importantly, there was no main effect of posture for either surface, but a significant interaction between attention and posture. This effect was selective for the alpha band when emphasizing the external reference frame. This effect was also similarly distributed across channels for hand and tool, with Bayesian analyses identifying positive evidence that they reflect the same processes. Furthermore, source localization of this effect for both surfaces revealed that comparable cortical networks were involved. Overall, these findings provide evidence that similar neurocomputational mechanisms are used by the brain to process touch location on the hand and on a hand-held tool. These mechanisms are reflected by alpha activity when manipulating posture, suggesting the use of external coordinates.

### Touch on hands and tools relies on shared oscillatory mapping mechanisms

The main result of this study is that localizing touch on hands and tools involves similar oscillatory correlates. Indeed, irrespective of coding in external and anatomical coordinates, not only were no significant difference found between oscillatory power of alpha and beta band between surfaces (see Table 2) but also most notably their scalp topographies were almost identical between hand and tool when observed separately (Figures 3A and 3B for alpha; Figure S1). These results appear to be consistent with the centuries-old proposal of tool embodiment.^1^ Incorporation of a hand-held tool into body representation may indeed consist in repurposing the neural mechanisms that process body-related sensory information for processing information originating from the tool. Until now, neuroscientific evidence for this proposition has been scarce, since the majority of evidence comes from behavioral studies and from paradigms that only measure the effects that tool use induced on subsequent perceptual or motor measures. For example, initial evidence of online repurposing comes from Iriki and colleagues’ work^12^ who measured from macaque monkeys’ multisensory postcentral neurons during tool use and observed an expansion of the visual portion of their receptive field to encompass the tool. At the behavioral level, online remapping of space (i.e., space previously coded as far being remapped as near) was observed during tool use by Berti & Frassinetti,^9^ but their neuropsychological approach could not provide indications as to which mechanisms are at play. The present study overcomes this limitation by measuring oscillatory activity underlying spatial coding for touch on hands and tools.

Our findings are consistent with previous studies on the neural correlates of tool sensing. We previously recorded EEG activity of human participants during a tactile localization task on a hand-held tool, therefore directly observing online tool use. We identified a modulation of alpha power dependent of contact location,^14^ suggesting that it is a signature of tool-extended tactile localization. Consistent with these previous results, here we also found that posture modulated alpha activity dependently of attention: for both surfaces, interaction effects were localized in two parieto-occipital clusters, one in each hemisphere (Figures 3A and 3B). We found some differences in the distribution of interaction effect of Attention and Posture between Surfaces. The right occipital cortex was notably activated for the hand, whereas the inferior temporal cortex was activated for the tool.^38^ This could be explained by the effect of attention on actively shaping and enhancing spatial representations in the ventral visual pathway.^39^ Besides this difference, the source reconstruction of the alpha modulation was largely comparable between surfaces (Figures 3C and 3D). This new evidence adds to our previous event-related potentials (ERP) study^13^ on tool-extended sensing: touches on the tool and on the arm led to similar stages of cortical processing as well as similar sources involved.

In sum, the remarkable similarity that we found for oscillatory processes for tactile localization on the hand and on the tool suggests that, in order to localize a contact happening on a hand-held tool, the human brain repurposes neural mechanisms dedicated to body-related processes to perform the same function with a tool.

### Alpha rhythm reflects external spatial coding for touch on hands and tools

Crossing limbs is a well-established method to tease apart localizing processes in external and skin-based coordinates. In this respect, previous electrophysiological studies have linked alpha oscillations to a use of external coordinates and beta to skin-based coordinates. Here, we manipulated hands and tools posture to characterize and compare the cortical oscillations reflecting the crossing effects emerging from hands and tools. This is especially of note for the tool, since only the tool tips crossed the body midline while the hands stayed in their respective hemispace (see Figure 1A). Therefore, any effect observed for crossing when touch is on the tool surface would reflect the mapping of touch on the tool, not the hands.

Consistent with previous findings,^25^ we observed that modulation of alpha activity following posture change was dependent on attention in external space, which was not the case for beta, supporting the involvement of the alpha band in external processing. Crucially, this modulation of the alpha activity was independent of whether touch localization processes concerned the hands or the tools, as exemplified in the near-identical scalp topographies and significant posterior clusters for both surfaces (Figures 3A and 3B). Our previous study^14^ also found an involvement of alpha oscillations in the encoding of touch location on tools. While this suggested the encoding of an external spatial code, the paradigm we used did not manipulate posture and was therefore equivocal in these regards. However, the present results indeed support the proposition made by Fabio et al.^14^ that touch on a tool is primarily coded in an external reference frame.

Furthermore, encoding touch localization in external coordinates on the hand and on the tool involves a similar cortical network. Source reconstruction of the alpha coding of external space identified several regions throughout the parietal and frontal cortices in both hemispheres. This included primary somatosensory and motor cortex. Importantly, the posterior parietal sources, also shared by the two surfaces, have previously been implicated in the processing of touch in external space.^40^^,^^41^^,^^42^^,^^43^ Parietal alpha oscillatory activity indeed appears to play a crucial role in this process.^24^^,^^25^^,^^27^

Complementary results were obtained in our analysis of tactile localization in anatomical coordinates. Consistent with prior findings, beta desynchronization was related to coding the hand or tool that was touched. However, the scalp distribution and sources suggest that this coding is independent of which surface was touched. To summarize, we found that alpha band indexes the spatial coding of touch in an external reference frame whereas the beta band indexes spatial coding in anatomical coordinates. Furthermore, this effect is independent of whether touch was localized on the hand or a hand-held tool.

### Alpha-based coding of external coordinates may depend on attention

Unsurprisingly, we found attention to modulate the overall oscillatory activity of both alpha and beta bands^33^^,^^44^^,^^45^ (see Table 1). However, it is noteworthy that posture manipulation itself was not sufficient to affect oscillatory activities; the modulation of alpha power following the crossing of the hands/tools was dependent on attention. This finding suggests that external processing of touch was dependent of certain attentional processes. Alpha oscillations have indeed been implicated in tactile spatial attention.^33^^,^^34^^,^^35^^,^^36^ Post-touch alpha oscillations may thus reflect the orienting of attention in external space.^46^

This involvement of attentional processes in spatial coding was reflected in the scalp topographies and source localization of our interaction effect, which was mostly localized in somatosensory^36^^,^^47^ as well as posterior regions of the cortex, for the hand as well as the tool (Figure 3). This pattern of results is fitting with previous studies about the modulatory effects of attention on tactile ERP.^48^^,^^49^ In particular, Eimer et al.^50^ suggested that different spatial coordinate systems may be used by separable attentional control processes with a posterior process operating on the basis of external spatial coordinates, whereas an anterior process is based primarily on anatomically defined spatial codes. Since crossing the hands (and hand-held tools) mainly modulates the external coordinates of tactile processing, our tactile spatial localization task likely involved spatial attentional processes taking places in external coordinates. Along these lines, Yue et al.^51^ also found that ERPs-to-tactile stimuli presented at the tips of tools were modulated by spatial attention when using a similar experimental paradigm.

To conclude, we found that the brain uses similar oscillatory mechanisms for mapping touch on a hand-held tool and on the body. These results are in line with previous work and support the idea that neural processes devoted to body-related information are being reused for tool use. Furthermore, alpha band modulation followed the position of touch into external space. This is thus the first neural evidence that tactile localization on a hand-held tool involves the use of external spatial coordinates.

### Limitations of the study

The present study aimed to compare the oscillatory underpinnings of spatial coding for touch on hands and hand-held tools. Whereas we conclude that the underlying mechanisms are similar for both surfaces, we acknowledge that there are methodological and conceptual limitations to our study. First, the interpretation of similar mechanisms, though predicted, relies on a null result, namely, the lack of an interaction between hand and tool conditions. While null results should be approached with caution, given the highly convergent pattern observed in alpha-band activity for the both surfaces and, most important, the confirmative result of the Bayes statistics, we consider this to be a minor limitation. Second, while we can conclude that alpha is tied to processing touch in external coordinates, the actual function of alpha (for both hands and tools) remains a mystery, one that we cannot, and did not, aim to solve in this study. Third, our findings only provide correlational evidence in favor of the role of alpha oscillations in external spatial coding. While this limitation is inherent to the electrophysiological measures we employed, future work should be devoted to causally link alpha-band activity to the remapping of spatial codes (usually referred to as tactile remapping) on hands and tools. Finally, it is important to note that the spatial resolution of source reconstruction is limited, especially for time-frequency decomposition. The precise localization of the implicated regions is therefore challenging to determine accurately. We therefore took caution when interpreting the source reconstruction, choosing to refrain from speculating about the computations performed by individual areas in our significant clusters. Furthermore, this limited spatial resolution also makes it impossible to distinguish between actual neuronal populations. It is possible that differences between how touch on hands and tools is implemented are only visible at a finer level of spatial resolution.

## STAR★Methods

### Key resources table

**Table:** 

REAGENT or RESOURCE	SOURCE	IDENTIFIER
**Deposited data**		

EEG data	OSF	https://osf.io/3r5jy/

**Software and algorithms**		

MATLAB software	Mathworks	https://fr.mathworks.com/products/matlab.html
EEGLab Toolbox	Delorme and Makeig^52^	https://doi.org/10.1016/j.jneumeth.2003.10.009
Brainstorm toolbox	Tadel et al.^53^	https://doi.org/10.1155/2011/879716

### Resource availability

#### Lead contact

Further information and requests for resources and reagents should be directed to and will be fulfilled by the lead contact, Luke Miller (luke.miller@donders.ru.nl).

#### Materials availability

This study did not generate new unique reagents.

#### Data and code availability

• EEG data have been deposited on an OSF repository and are publicly available as of the date of publication. OSF repository is listed in the key resources table.
• This paper does not report original code
• Any additional information required to reanalyze the data reported in this paper is available from the lead contact upon request.

### Experimental model and study participant details

#### Participants

20 right-handed participants (mean age: 24.8 years; range: 18–32 years, 10 males), free of any known sensory, perceptual, or motor disorders, volunteered to participate in the experiment. We chose this sample size in accordance to previous studies about somatosensory oscillations. The experiment was performed in accordance with the ethical standards laid down in the Declaration of Helsinki (2013) and all participants provided written informed consent according to national guidelines of the ethics committee (CPP SUD EST IV RCN: 2010-A01180-39).

### Method details

#### Setup

The experiment was divided in two sessions wherein touch was delivered to either the participants’ hands (Hand condition) or to hand-held wooden rods (Tool condition); the setup was similar for both conditions. Throughout the experiment, participants sat in an adjustable chair in front of a table. In the Hand condition, they placed their forearm on the table, positioned either in an uncrossed or in a crossed posture (alternated blockwise, order counterbalanced across participants) with each index finger resting on a support at the edge of the table (Figure 1A). In the Tool condition, participants’ arms were placed on adjustable armrests and they hold a 50cm long wooden rod in each hand, either in an uncrossed or in a crossed posture (alternated blockwise, order counterbalanced across participants). Notably, in the Tool condition, only the tool-tips -but not the hands-crossed their body midline (Figure 1B)—the overall posture of the upper limb was relatively unchanged when tools were crossed or uncrossed. Depending on the condition, the index fingertips or the tip of each tool rested on a foamed stabilizing support at the edge of the table. When crossed, the rods were raised one above the other in order to avoid their contact during stimulation.

Participants fixated on a central cross (2 cm wide) that was displayed on a 16″ monitor in front of them and aligned with their body midline. Two solenoids (Mecalectro 8.19-.AB.83; 24 V, supplied with 36 W) were used to contact either index fingers or tool-tips, both with a disc surface of 4 cm to ensure uniform and consistent contact. Behavioral responses were made with a foot pedal (Leptron Footswitch 548561) that was placed underneath the left foot in half of the experiment, and under the right in the other half (alternated condition-wise, order counterbalanced across participants).

We took two approaches to mitigate potential auditory spatial cueing from the solenoids. First, noise-cancelling earphones (Bose QuietComfort 20) playing white noise were used to mask the sound created by the solenoids. Further, to avoid any remaining auditory cue, each solenoid had another (decoy) solenoid placed on the opposite side, not in contact with the participants’ hands or tools, that was activated synchronously. All solenoids were mounted on adjustable tripods. Visual feedback was prevented by covering the table with a white cardboard.

#### Experimental paradigm

Participants performed a tactile discrimination task divided in two sessions depending on the surface stimulated (Hand or Tool). In this task, participants were cued to pay attention to one side of external space. They then had to identify a deviant stimulus (double tap) presented to a hand/tool in this cued side, while ignoring stimuli in the uncued side of space. Importantly, posture was manipulated to disentangle the involvement of different reference frame transformations. Each participant completed both sessions of the experiment on separate days (counterbalanced). Each session started with a practice block of 42 trials in both postures (Uncrossed and Crossed) to ensure they correctly understood and complied with task instructions.

At the beginning of each trial the central cross blinked to indicate the start of the trial. Then one side of the cross briefly turned blue (for 50 ms) to indicate which side of space (left or right, equal probability) participants had to attend to (Figure 1C). After a variable delay (between 1000 and 1500 ms; randomly chosen from a uniform distribution), tactile stimulation was applied on participants’ right or left finger (Hand session), or on the tip of the right or left rod (Tool session). Note that this was independent of the cued side. Tactile stimuli were either frequent standard stimuli (solenoid raised once for 50 ms, including rise time and surface contact; probability of 0.75), or rare deviant stimuli (solenoid raised twice in a row for 50 ms separated by a 75 ms gap; probability of 0.25) presented with an equal probability in a random sequence to the left and the right. Participants had to respond as fast and accurately as possible using the foot pedal to rare tactile deviants presented to the cued side (“targets”, probability of 0.125), and to ignore standard stimuli at the attended side, as well as all stimuli presented to the other side. Each trial lasted between 2750 and 3750 ms. The experiment consisted of two sessions of 10 blocks, half Crossed, half Uncrossed. Each block included 60 standard trials and 20 deviant trials. The analysis included only trials in which standard stimuli were presented and in which, accordingly, no response was required, for a total of 600 trials per sessions. Participants complied with instructions, as evidenced by their high accuracy in each posture and for both surfaces (all>96%, see Table 1).

#### EEG recording

EEG data were recorded continuously using a 65 channel ActiCap system (Brain Products). Horizontal and vertical electro-oculograms (EOGs) were recorded using electrodes placed below the left eye, and near the outer canthi of the right eye. Impedance of all electrodes was kept at <20 kΩ. FCz served as the online reference. EEG and EOG signals were low-pass filtered online at 0.1 Hz, sampled at 2500 Hz, and then saved to disk. Stimulus presentation and behavioral response collection were performed using MATLAB on the experimental control computer, which was synchronized and communicated with the EEG data recording system. The trial events were sent to the EEG data recording system via a parallel port.

#### Pre-processing of the EEG data

EEG signals were preprocessed using the EEGLab Toolbox.^52^ The preprocessing steps for each participant were as follows: for each session, participants’ five blocks in uncrossed posture, followed by their five blocks in crossed posture, were appended into a single dataset. The signal was resampled at 500 Hz and high-pass filtered at 0.1 Hz. Faulty channels were interpolated using a spherical spline. We then epoched data into a time window of 3.5 s, 1 s before and 2.5 s after the cue and baseline corrected using the period from −500 ms to −100 ms before the cue as baseline. Next, we removed signal artifacts with two steps: first, we removed eye blinks and horizontal eye movements from the signal using independent components analysis (ICA^52^) and a semi-automated algorithm called SASICA.^54^ We excluded every trial when participants made a response or accidentally released the foot pedal, so that we only kept correct standard trials that were free of any motor activity. We re-epoched the data around the tactile stimulation (time zero), from −1.5 s before to 1 s after, leading to 2.5s epochs, and used the period from −500 ms to-100 ms before contact for baseline correction. We then manually rejected trials that were contaminated by muscle artifacts or other forms of signal noise. In total, this led to a mean exclusion of 52.6 trials per participant (range: 2–205). Next, we used the EEGLab function *pop_reref* to add FCz (the online reference) back into the dataset and re-referenced the data to the average voltage across the scalp. Finally, for a better signal-to-noise ratio, we remapped the electrodes channels to ipsi- and contralateral recording sites relative to the touched hand or tool. Therefore all trials would now be in reference to the right hand or tool being touched.^23^^,^^25^

### Quantification and statistical analysis

#### Time-frequency decomposition

Time-frequency decomposition was performed using the open source toolbox Brainstorm^53^ in MATLAB. The raw signal of each epoch was decomposed into frequencies between 1 and 35 Hz (linearly spaced) using Complex Morlet wavelets with a central frequency of 1 Hz and a full-width half maximum of 3 s. These parameters were chosen to ensure that our time-frequency decomposition had good spectral and temporal resolution within the chosen frequency range. Signal was then normalized in decibel (dB) to its ratio with the respective channel mean power during a baseline period ranging from −500 to −100 ms before tactile stimulation, which ensured that no post-stimulus activity contributed to the baseline normalization.

#### EEG data analysis

Based on the observation of the oscillatory temporal dynamics during the time period after contact (collapsed across all participants and conditions, see Figure 1D), we defined alpha- and beta-band frequency range here as 8–13 Hz and 15–25 Hz respectively. We also selected two time windows for analysis for each of our frequency band of interest: 150–300 ms for beta-band, and 250–500 ms for the alpha-band. This is a bias-free method for choosing time windows upon which running the analysis.^55^

Our experimental design had three factors that could be used in our analysis: Surface (Hand or Tool), Posture (Uncrossed or Crossed) and Attention (Unattended or Attended). Our initial analysis included all factors. The main effect of each factor was calculated by averaging the power of each frequency band, as well as the time points within the given time window before comparing the scalp topography of the relevant levels (e.g., Uncrossed vs. Crossed for main effect of Posture) using a cluster-based permutation test^56^ (two-tailed, cluster-level significance threshold of 0.05 and 1000 permutations run). Interaction effects were assessed via subtraction across conditions. For example, take the three-way interaction between Attention, Posture, and Surface. We first calculated the differences between unattended and attended stimulation for each posture separated by surface (e.g., Hand crossed unattended – Hand crossed attended). We then subtracted these differences for each surface before comparing them for each frequency band (average power) in their respective time windows (average time points; CBPT, same parameters). For all two-way interactions (e.g., Attention x Posture) we first collapsed across the unused condition (Surface); we then calculated the difference between levels of the first factor (Unattended – Attended) for each levels of the second factor (Uncrossed, Crossed). In a secondary analysis, we analyzed both surfaces separately.

To more directly test the lack of a difference between hand and tool, we conducted Bayesian paired t-tests (JASP; default prior) on each surface’s Posture × Attention interaction contrast. Specifically, the analysis was conducted on the average signal within each significant cluster found in the Posture × Attention interaction of the omnibus ANOVA. We used the heuristic of a Bayes Factor (BF) > 3 indicating a significant effect, with BF_10_ > 3 corresponding to significant evidence *in favor* a difference between tool and hand and BF_01_ > 3 corresponding to significant evidence *against* a difference between tool and hand.

The current study focuses on the effect of posture on the processing of touch location (i.e., post-stimulus processing). It is therefore important that any effects we observe are not due to the baseline period before the contact. We therefore also analyzed oscillatory activity in the cue target interval (600–1000 ms post-cue; i.e., overlapping with our chosen baseline) in order to uncover potential differences in the orienting of attention across conditions. We did not find any significant effect of posture or spatial orienting on alpha and beta power during this period. We can therefore be confident that the above analysis reflects post-contact spatial localization.

#### Source reconstruction

We followed up significant interactions with source reconstruction for each epoch to estimate which brain regions were involved. This was done using the open source toolbox Brainstorm.^53^ First, a head model was computed using OpenMEEG BEM model.^57^ A noise covariance matrix for every participant was computed over a baseline time window of −500 to −100 ms before stimulation. Sources were then estimated using the Standardized low resolution brain electromagnetic tomography (sLORETA^58^) approach with unconstrained dipole orientations across the surface. We then performed time-frequency decomposition on the source files to localize significant power modulations in the alpha-band. The signal at each vertex was decomposed into the mean of frequencies going from 8 to 13 Hz using Complex Morlet wavelets with a central frequency of 1 Hz and a full-width half maximum of 3 s. The signal was then normalized in decibel (dB) to its ratio with the respective channel mean power during a baseline period ranging from −500 to −100 ms before tactile stimulation.

The interaction between Attention and Posture was assessed in a similar manner as previously described. We calculated the difference in alpha power between unattended and attended stimulation for each posture and compared them in the chosen time windows (average time points) using Cluster-based permutation^56^ (two-tailed, cluster-level significance threshold of 0.05 and 1000 permutations run).
