# Supplementary material for: Alpha oscillations reflect similar mapping mechanisms for localizing touch on hands and tools
Source: iScience. 2024 Feb 2;27(3):109092. doi: 10.1016/j.isci.2024.109092 (PMC10884914; doi:10.1016/j.isci.2024.109092)
Supplement: Document S1. Figure S1 [file mmc1.pdf]

iScience, Volume *27*

Supplemental information

Alpha oscillations reflect similar mapping mechanisms for localizing touch on hands and tools

Cécile Fabio, Romeo Salemme, Alessandro Farnè, and Luke E. Miller

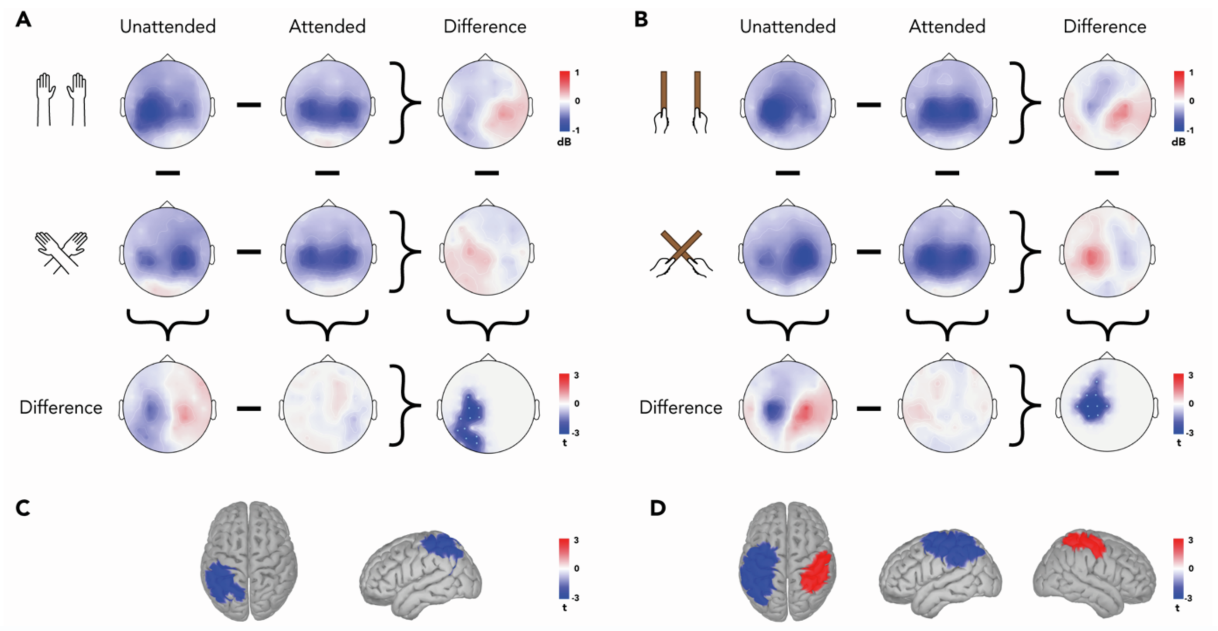

Supplementary Figure 1. Beta activity following tactile stimulation on the hand and on the tool, related to Figure 4
(A) Topographies of beta-band activity (15-25Hz, 150 to 300ms) when tactile stimuli happened on the hand, with uncrossed (1st row) and crossed (2nd row) hands following attended (1st column) and unattended (2nd column) stimuli. Difference topographies for attention effects with uncrossed and crossed hands (3rd column), and for posture effects following attended and unattended stimuli (3rd row). Bottom-right corner: topography of the interaction between attention and posture. (B) Topographies of beta-band activity (15-25Hz, 150 to 300ms) when tactile stimuli happened on the tool, with uncrossed (1st row) and crossed hands (2nd row) following attended (1st column) and unattended (2nd column) stimuli. Difference topographies for attention effects with uncrossed and crossed tools (3rd column), and for posture effects following attended and unattended stimuli (3rd row). Bottom-right corner: topography of the interaction between attention and posture. (C) Source reconstruction of the interaction effect between attention and posture for tactile stimulation on the hand. (D) Source reconstruction of the interaction effect between attention and posture for tactile stimulation on the tool. Data are displayed as if stimuli always occurred on the hand or tool localized in the right hemispace, independent of posture.
